# Closing the Implementation Gap: Comment on "Insights Gained From a Re-analysis of Five Improvement Cases in Healthcare Integrating System Dynamics Into Action Research"

**DOI:** 10.34172/ijhpm.2022.7593

**Published:** 2022-11-01

**Authors:** Sally Brailsford

**Affiliations:** Southampton Business School, University of Southampton, Southampton, UK

**Keywords:** Simulation, Implementation, System Dynamics, Action Research

## Abstract

Holmström et al provide an interesting and thought-provoking contribution to a perennial problem: why, despite a vast number of applications of simulation modelling in healthcare over the past 70 years, there is still remarkably little evidence of successful implementation of model results. Their paper is a retrospective analysis of five case studies, all undertaken as consultancy, that used a blend of system dynamics (SD) modelling and action research (AR). This commentary assesses the effectiveness of this approach in achieving implementation, based on the evidence presented, and discusses some of the issues raised. These issues include a comparison of Holmström’s approach with group model building (GMB) in SD, the differences between healthcare modelling projects undertaken by (*a*) business consultants and (*b*) academics, and the challenges of undertaking ‘systematic’ reviews of the grey literature.

 Simulation modelling has been widely used in healthcare applications since the 1960s, both by academics and by commercial business consultancies. However, despite a plethora of survey papers over the decades (Tunnicliffe Wilson,^1^ Lagergren,^2^ Fone et al,^3^ Brailsford et al,^4^ Tako and Robinson,^5^ Darabi and Hosseinichimeh,^6^ Carter and Busby^7^ to name but a few) there is scant evidence in the literature of such models having much impact on practice. The figure of 5% for ‘implemented’ system dynamics (SD) models cited by Holmström et al^8^ derives from Brailsford et al^4^ and refers to journal articles that state that the model findings are being used by a healthcare organisation. However, a depressingly tiny fraction of this 5% actually report the outcomes following implementation, and the vast majority of published articles do not mention implementation at all. One reason for this is that simple models or standard applications of known methods are more likely to be used in practice, yet papers that describe such models are less likely to be accepted for publication. Journal editors normally make decisions based on the scientific or technical novelty of the content, not whether the results are useful.

 The key point is that all these survey papers are based solely on the academic literature. While some academics also undertake consultancy, and the boundary between applied research and consultancy can be blurred, business consultants tend not to publish in academic journals. This is not only because of the requirement for a ‘scientific contribution’ but also because there are no commercial incentives for them to do so. Publication is often a lengthy process and articles may be hidden behind paywalls, rather than readily visible to potential clients in the so-called ‘grey’ literature (websites, newsletters, blogs, social media, promotional material, public domain reports, conference presentations, and so on). Brailsford et al^4^ state:

 “*The challenge is to find a viable means of accessing and referencing these sources, which by definition are not recorded in conventional bibliographic databases. Despite this we believe that ‘grey literature’ may be centrally important in revealing lessons to be learned from the implementation of models in healthcare, an area that seems to be sorely absent in most of the research literature reviewed here”*^4^ (p. 139).

 There is still no universally recognised and rigorous methodology for searching the grey literature, compared with the established methodologies for conducting systematic reviews of the academic literature. Hence it remains difficult to estimate the proportion of implemented models developed by business consultants for healthcare clients. However, it is likely to be higher than 5%, if only because senior hospital managers may be reluctant to admit to their governing boards that they spent money on consultancy and then ignored the resulting recommendations! On the other hand, business consultancies are unlikely to publicise their unsuccessful projects, whereas academics have different criteria for judging success; an innovative mathematical model can get published in a top journal even if no one ever uses it in practice.

 Holmström et al^8^ present an interesting and thought-provoking contribution to this thorny implementation problem. While the paper has been published in a respected academic journal, and undoubtedly constitutes research, the case studies it presents and reflects on were undertaken as consultancy by a business consultant. In a sense, therefore, it represents a bridge between the worlds of academia and consultancy (and their respective literatures). The authors claim that their chosen blend of action research (AR) and SD modelling increases the likelihood of successful implementation, and provide evidence for this through five case studies where this approach was used. Their argument is based on the hypothesis that AR is a highly effective way to achieve a solution that is acceptable to all stakeholders and works in practice, but the cycle of understanding the problem, generating candidate solutions, testing them in the real world, potentially modifying them in the light of experience, and then starting again can be both time-consuming and expensive. Hence using computer simulation (in this case, SD) in the testing phase can speed up the process considerably.

 In the social sciences, AR is normally understood as a research philosophy where the researcher is also a participant, ie, conducts research ‘with’ people rather than ‘on’ them: for example, the researcher may become a temporary employee of the studied organisation. The aim may be (as in these five case studies) to solve a specific problem, or more broadly to gain insight into individual or organisational behaviour. Psychologically, in AR the researcher aims to be perceived as ‘one of us’ by the study participants. There is clearly a different dynamic when the researcher is a business consultant whose time is paid for by the client organisation, compared with an academic whose time is normally paid for by someone else, although both may be treated with suspicion (sometimes unfairly, sometimes not!) by the other participants. AR is essentially a philosophical approach rather than a methodology and as Holmström et al point out, many different techniques, both quantitative and qualitative, can be used to conduct the actual research. It is therefore not strictly accurate to call the approach used in these five case studies, where SD is embedded within an AR framework, a ‘mixed methods’ approach.

 There are many similarities between Holmström’s approach and group model building (GMB) in SD.^9^ GMB is founded on the belief that a participatory process fosters a shared understanding of the problem to be addressed, while engagement of all stakeholders engenders confidence in the model and a common ownership of the solutions that emerge. The authors (correctly) state that the approach adopted in these five case studies is distinct from scripted GMB, but in my view it has a lot in common with the unscripted form of GMB, ie, where the facilitated sessions are flexible and bespoke to the problem setting; while all of Holmström’s five case studies had the same broad structure, the steps were not always executed in exactly the same order. Both approaches involve cycling through a number of iterations until a final solution is reached, and both involve a number of meetings over a period of time. In Holmström’s five case studies, there were four or five meetings per case. In both approaches it is important that (as far as possible) the same people attend each meeting. In my personal experience this can be challenging to achieve in healthcare, although it may be easier if the collaborating/client organisation is paying consultancy rates! Even so, in case 1, one (powerful) clinical stakeholder never attended any meetings. It was also a little surprising to see that despite ‘patient centred care’ being a key element in most of the causal loop diagrams, the patient voice was missing from all five case studies: none of the groups contained any patients as participants.

 Although Holmström et al state that GMB participants need to learn how to develop quantitative SD models themselves, which would mean that Holmström’s approach is more accessible than GMB, in practice this is rarely the case. In the majority of real-world applications of GMB, as in these five case studies, participants only need to learn the basic principles of causal loop diagramming; any hands-on computer modelling is undertaken by an expert.^10^ In Holmström’s approach participants definitely need an elementary understanding of stock-flow models, or at least of interpreting the results, as all five cases involved the development of a quantitative model that was used in meetings to conduct ‘what if’ experiments.

 This raises the wider issue of whether it is better, in general, for the facilitator to do the computer modelling or whether the roles should be separated. There are arguments in favour of both approaches and obviously it also depends on the skillset of the facilitator. Holmström et al provide a fascinating and insightful discussion of the role of the facilitator in both approaches, and the skills required. In GMB it is fairly common for one person to build the model (live, in meetings) while a second facilitates the discussion. This allows the facilitator to be a trusted ‘comforter’ to the participants (in Holmström and colleagues’ words) and act as an interface between the group and the modeller. Modellers, especially if they are academics, can sometimes be perceived as technical ‘geeks’ who are expert in using computer software but have limited understanding of the real world situation. The closer the facilitator gets to being part of the group, rather than separate from it, the closer GMB gets to AR. It is interesting to note that while Holmström acted as both modeller and facilitator, he did most of the actual computer work between meetings.

 Holmström et al recognise that although their analysis of these case studies uses a consistent framework, based on the stages of consultancy projects, it is retrospective and may be subject to recall bias. This is a common limitation in any evaluation. In their analysis of the success of 107 GMB interventions where an outcome was reported, Rouwette et al^10^ comment that only three of these involved a before-and-after survey of participants, and the vast majority (80%) described qualitative case studies that were largely based on observational data. The pressure on academics to ‘publish or perish’ can lead to papers being written and submitted before there has been time for a collaborating organisation to implement the model findings, let alone conduct a proper evaluation. Moreover, in healthcare, given the complexity of the system and the rate of organisational change, successful implementation can often be determined by political or external factors beyond the control of the ‘client,’ no matter how enthusiastic they are about the model and its results.^9^

 The reader is not told whether any other projects using this combined AR/SD approach turned out to be less successful than these five... and there is always a question of determining the optimal time to conduct an evaluation. The case studies were undertaken over ten years ago, between 2004 and 2011, and while Holmström et al report the short-term outcomes of each project (and also what participants said about them at the time, which was generally positive), the longer-term outcomes are only reported for case study 2. The findings of this project were *“…accepted and tested for a month after which minor changes were made before the final implementation, which was evaluated showing good results*” but none of the other four appear to have been evaluated. Case study 5 states “*Some years later the department received entirely new and larger facilities after having done extended studies” *but it is not clear whether these extended studies were based on the initial modelling work, or were unrelated.

 In summary, this paper is undoubtedly an interesting contribution to the literature on the implementation of simulation modelling in healthcare. As briefly discussed at the end of the paper, the idea of using other simulation approaches, ie, discrete-event or agent based simulation, in combination with an AR based approach looks very promising. Although it would be time-consuming, this is probably unavoidable if a successful outcome is to be achieved. One of the (many) barriers to implementation in healthcare is the ‘not invented here’ problem,^11^ ie, it is simply not possible to bypass the lengthy process of stakeholder engagement in model building and reuse a model that was developed for a different client, no matter how similar the problem setting. “*All modellers stress the importance of involving the client/end user at every stage of model development, as being the only way to secure buy-in*”^11^ (p. 314). Genuine AR would take an extreme approach to this by fully integrating the modeller into the client/end user organisation, but the modified AR approach proposed by Holmström et al represents a pragmatic compromise.

## Ethical issues

 Not applicable.

## Competing interests

 Author declares that she has no competing interests.

## Author’s contribution

 SB is the single author of the paper.
